# Explantation of Two Embolized Balloon-Expandable Valves in Left Atrium After Transcatheter Mitral Valve Replacement

**DOI:** 10.1016/j.atssr.2024.11.009

**Published:** 2024-12-10

**Authors:** Andrew Marthy, Mei Zuo, Saeed Tarabichi, Eduardo Danduch, Sanjay Samy, Chikashi Nakai

**Affiliations:** 1 Department of Cardiothoracic Surgery, Albany Medical Center, Albany, New York

## Abstract

An 81-year-old man with a history of transcatheter aortic valve replacement for severe aortic stenosis presented with dyspnea on exertion and moderate-severe mitral stenosis. He underwent transcatheter mitral valve replacement (TMVR) through a transseptal approach, given his age and comorbidities. The TMVR procedure was complicated by 2 embolized TMVR valves into the left atrium, requiring urgent transfer to the operating room for exploratory cardiotomy. The embolized valves were explanted from the left atrium successfully without additional mitral valve intervention. Intraoperative transesophageal echocardiography revealed improved mitral stenosis from severe to mild level after TMVR valve deployments. He was discharged home on postoperative day 10.

Nonrheumatic mitral stenosis (MS), a condition that impedes transmitral blood flow, typically arises from degenerative processes or calcification.^1^ In an era of expanding transcatheter therapies, balloon valvuloplasty for severe disease followed by transcatheter mitral valve replacement (TMVR) has become more frequent.^2^ In addition, with increased rates of transcatheter aortic valve replacement (TAVR), patients may present with mitral valve disease after aortic valve intervention. Valve embolization is a critical complication of the TMVR procedure. In this situation, emergent surgical intervention is most often required.^3^

An 81-year-old man who had TAVR with a 26-mm Sapien3 valve (Edwards Lifesciences) for severe aortic stenosis previously presented with dyspnea on exertion. Past medical history included nonrheumatic MS, coronary artery disease, atrial fibrillation, and chronic heart failure. Transthoracic echocardiography (TTE) showed preserved left ventricular ejection fraction and moderate-severe MS with severely reduced mitral valve leaflet mobility, mean pressure gradient (mPG) of 9 mm Hg, mitral valve area of 1.4 cm^2^, and right ventricular systolic pressure of 54 mm Hg. Stress TTE demonstrated mPG of 11 mm Hg, consistent with severe MS, and elevated right ventricular systolic pressure of 76 mm Hg. By a heart team approach, the patient was at prohibitive operative risk because of comorbidities, and a transcatheter intervention was pursued. The patient underwent TMVR through a transseptal approach. The first 29-mm Sapien3 valve was deployed at the mitral position successfully. However, intraprocedural transesophageal echocardiography (TEE) revealed migration of the valve toward the left atrium (LA; Figure 1A) and a significant perivalvular leak (Figure 1B). The decision was made to proceed with a second valve-in-valve procedure with a 29-mm Sapien3 valve (Figure 2A). There was a residual perivalvular leak after the second valve deployment. Postdilation was performed for the perivalvular leak (Figure 2B). After postdilation, both valves embolized into the LA (Figure 2C, 2D), requiring urgent transfer to the operating room for exploratory cardiotomy with sternotomy. Under cardiac arrest, an incision into the dome of the LA was made and extended posterior caudally. Both valves were retrieved from the LA. Visualization of the native mitral valve was impossible for the preexisting TAVR valve in the aortic position. Intraoperative TEE revealed only mild MS with mPG of 3 mm Hg; therefore, mitral valve intervention was not performed. The postoperative course was complicated by respiratory failure, which was resolved with medical management. Follow-up TTE on postoperative day 2 confirmed mild MS with mPG of 5.5 mm Hg and mitral valve area of 1.8 cm^2^. The patient was discharged home on postoperative day 10.

**Figure 1:**
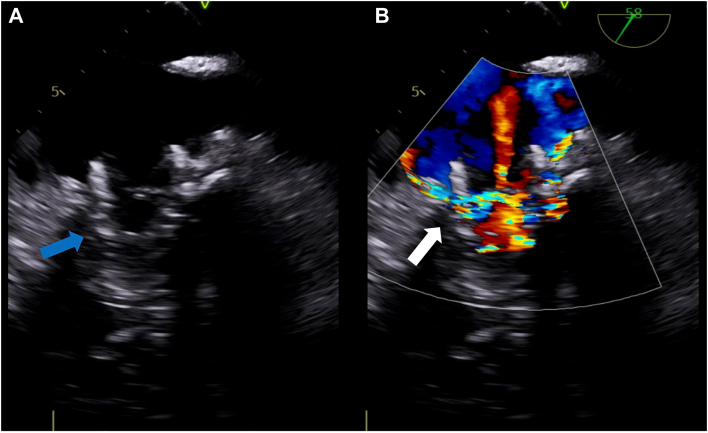
Intraprocedural transesophageal echocardiography after first valve deployment. (A) Transcatheter mitral valve replacement valve migrating toward left atrium (arrow). (B) Severe perivalvular leak (arrow).

**Figure 2:**
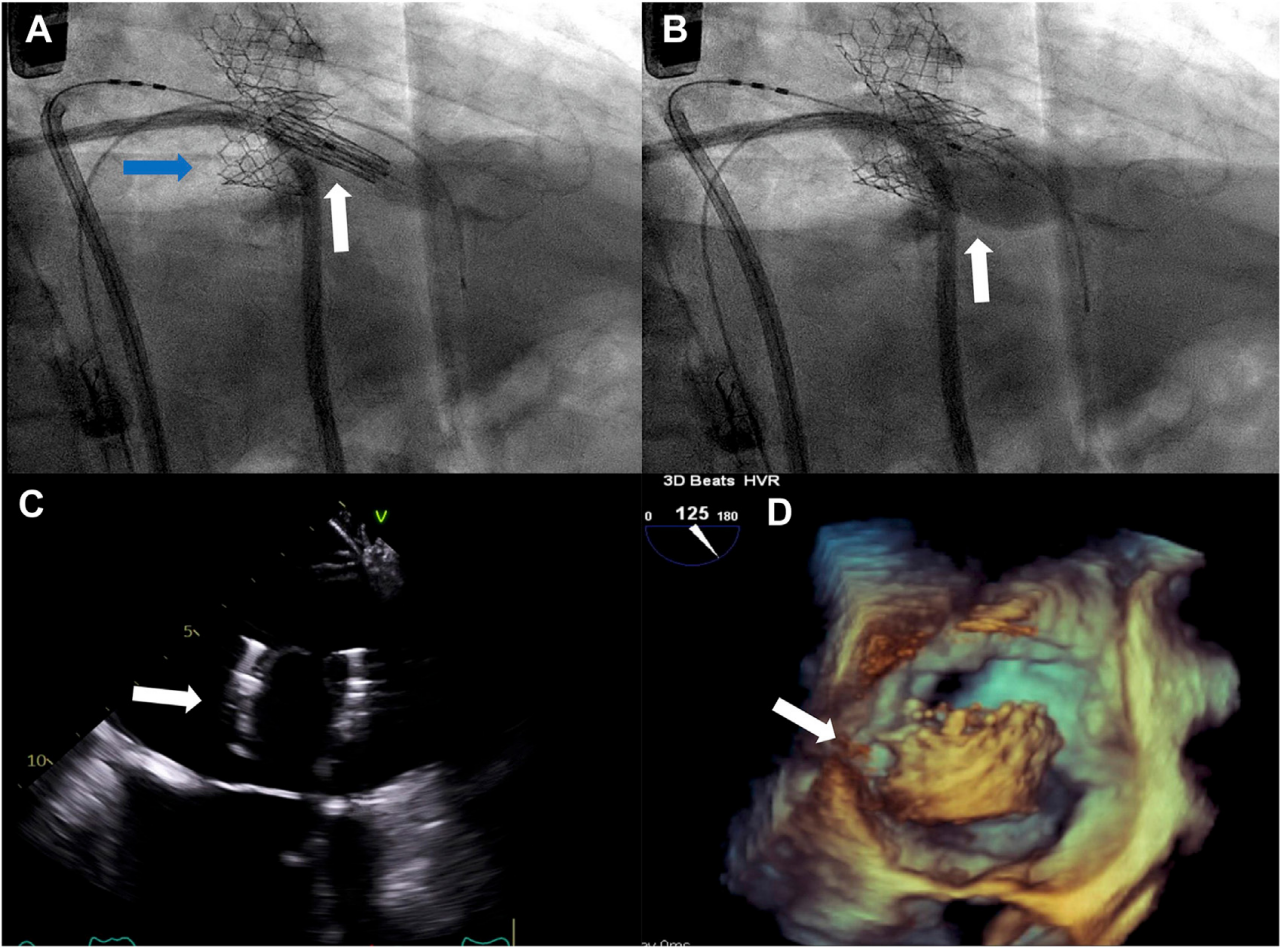
(A, B) Fluoroscopy in second valve delivery and deployment. (A) First valve in the mitral position but migration toward left atrium (blue arrow), second valve delivered in the valve-in-valve position (white arrow). (B) Deployment of second valve (arrow). (C, D) Intraprocedural transesophageal echocardiography after second valve deployment. Transcatheter mitral valve replacement valve embolized into left atrium (arrow).

## Comment

We experienced a case complicated by embolized TMVR valves into the LA in a patient status post TAVR. The patient was taken to the operating room emergently and underwent explantation of the embolized valve successfully without additional mitral valve intervention.

Valve embolization is a rare but significant complication of transcatheter valve replacement procedures and is associated with increased morbidity and mortality. The TRAVEL (TranscatheteR HeArt Valve EmboLization and Migration) registry, a multicenter, international collaboration with 26 sites, found that valve embolization occurred in approximately 1% of transcatheter aortic valve implantation cases.^4^ However, there are very few published cases of embolized valves after TMVR. Causes of valve embolization include suboptimal positioning, inadequate anchoring, manipulation, postdilation, and sizing error.^5^ Valve embolization often requires deployment of a second valve or conversion to open-heart surgical intervention.^6^ In our case, the mitral annulus calcification might not have been an adequate anchor; eventually, postdilation caused valve embolization. The treatment of valve embolization is complex, and emergent surgical timing may contribute to improved patient outcomes.

**Figure 3:**
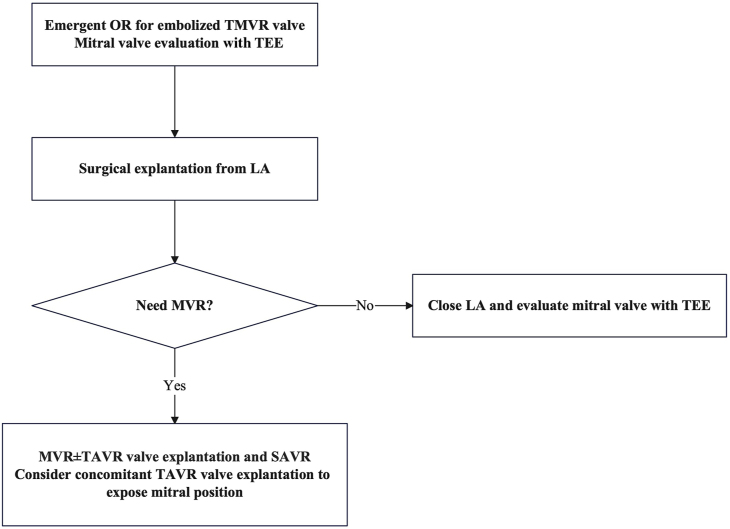
Management of transcatheter mitral valve replacement (TMVR) valve embolization in patient with status post transcatheter aortic valve replacement (TAVR) procedure. According to the requirement of a mitral valve replacement (MVR) procedure after embolized TMVR valve explantation, additional TAVR explantation and surgical aortic valve replacement (SAVR) should be considered. (LA, left atrium; OR, operating room; TEE, transesophageal echocardiography.)

In the transcatheter era, complications of valve implantation will likely increase. With the increasing use of TAVR in lower risks groups and in younger patients, the need for post-TAVR cardiac surgery will also become frequent. Mitral valve intervention is particularly relevant after TAVR as the implanted valve potentially interferes with exposure of the mitral valve for surgical mitral valve repair or replacement. Nonaortic valve surgery after TAVR is associated with increased mortality, with a recent series highlighting mitral valve intervention with an observed mortality of 13.5% and an observed to expected mortality of 1.8%.^7^ In patients who had surgical aortic valve replacement (SAVR) previously, the most implanted valves are stented bioprostheses with short stent frames.^8^ In contrast, transcatheter valve designs vary between manufacturers and may determine technical challenges encountered during TAVR valve explantation, including TAVR valve interaction with surrounding structures.^8^ With design variations of the TAVR valve, exposure of the mitral position in patients with a TAVR valve may be more difficult than with a SAVR valve. In our case, exposure of the mitral valve was challenging with a previous TAVR valve at the aortic annulus position. However, MS was improved from severe to mild level after the TMVR valve deployments. The decision was made to leave it alone without mitral valve intervention. Concomitant TAVR valve explantation and SAVR may be considered for exposure of the mitral position if the patient requires mitral valve replacement after explantation of a TMVR valve (Figure 3). For the decision to be made of whether mitral valve replacement is performed, intraoperative TEE plays a critical role for evaluation of the mitral valve (Figure 3). Simple balloon valvuloplasty might have been one of the options in our case, given the valve in mitral annulus calcification procedure and previous TAVR valve in the aortic position, to avoid perioperative complications. With increasing complexity of cardiac disease, including concomitant valvular disease, the heart team approach is optimally geared to address therapeutic interventions best suited for the patient. The heart team requires management for complications from transcatheter interventions to be established in advance, including valve embolization.
